# A recurrent *ACAA2* variant causes a dominant syndrome of lipodystrophy, lipomatosis, infantile steatohepatitis, and hypoglycemia

**DOI:** 10.1172/JCI198888

**Published:** 2025-11-04

**Authors:** Vinaya Simha, Mary Kate LoPiccolo, Anna Platt, Rebecca J. Brown, Xandria Johnson, Deanna Alexis Carere, Colleen Donnelly, Matthew T. Snyder, Chao Xing, Thomas P. Mathews, Purva Gopal, Stephen C. Ward, Diana R. Tomchick, Anil K. Agarwal, Ralph J. DeBerardinis, Abhimanyu Garg

**Affiliations:** 1 Mayo Clinic, Rochester, Minnesota, USA.; 2 Icahn School of Medicine at Mount Sinai, New York, New York, USA.; 3 University of Virginia, Charlottesville, Virginia, USA.; 4 NIH, Bethesda, Maryland, USA.; 5 UT Southwestern Medical Center, Dallas, Texas, USA.; 6 GeneDx LLC, Gaithersburg, Maryland, USA.

**Keywords:** Endocrinology, Genetics, Adipose tissue, Diabetes, Genetic diseases

## Abstract

We report a novel variant in *ACAA2* that causes hepatitis and hypoglycemia during infancy and lipodystrophy during adulthood accompanied by elevated plasma long chain acylcarnitines.

**To the Editor:** Familial partial lipodystrophies (FPLs) are rare genetically and phenotypically heterogeneous disorders characterized by symmetrical yet variable loss of subcutaneous fat from the extremities and trunk (1), often accompanied by abnormal fat accumulation in the face, neck, labia majora, and visceral depots. Previously, monoallelic variants in *LMNA*, *PPARG*, *AKT2*, *PLIN1*, *ADRA2A*, and *NOTCH3*, and biallelic variants in *CIDEC*, *LIPE*, and *PCYT1A* have been implicated in FPL (1, 2) (Supplemental Table 1; supplemental material available online with this article; https://doi.org/10.1172/JCI198888DS1). However, the molecular basis remains unknown in many individuals. Here, we report a heterozygous variant in the gene encoding acetyl-coenzyme A acyltransferase 2 (ACAA2), a mitochondrial fatty acid β-oxidation (mFAO) enzyme, in 4 families with FPL, lipomatosis, and variable occurrence of infantile steatohepatitis and hypoglycemia (IHH).

The main clinical features, individual case reports, pedigrees, and plasma levels of selected analytes from affected patients are in Figure 1, A and B, supplemental material, and Supplemental Table 2. All affected adults who were examined had marked loss of extremity fat, with excess fat in the neck and labia majora (Figure 1C and Supplemental Figure 1). During infancy, 3 had hypoglycemia, 1 had severe hypoglycemia-induced brain injury, and 6 had transient hepatitis; liver biopsies of 2 of these showed micro/macrovesicular steatosis, periportal fibrosis with bridging, and reduced mitochondrial cristae (Figure 1D).

Clinical exome sequencing (ES) of the FPL421 trio revealed a single heterozygous, de novo c.688G>A, p.Glu230Lys variant in *ACAA2* in the proband classified as “variant of uncertain significance.” Two years later, the same commercial laboratory identified this variant in another adolescent girl and her mother (FPL430) and in family IHH100 with 4 affected individuals. We then queried ES data from 257 unrelated, unresolved FPL patients at UT Southwestern and identified a fourth proband with this variant (FPL331.3). The identification of a de novo *ACAA2* variant in a proband and in 3 unrelated FPL families classifies it as strongly pathogenic (3).

ACAA2 has 397 amino acids and glutamic acid 230 is highly conserved (Figure 1, E and F) (4). The Glu230Lys substitution causes increased positive charge of the N-terminus of helix Lα4 and loss of the Glu230 to a Lys234 salt bridge (Figure 1G). These changes may enhance binding of coenzyme A (CoA) to the active site (Extended Results in supplemental material). Analysis of plasma acylcarnitines in 4 patients versus 8 controls revealed 2- to 35-fold increases in several long-chain (C14:0, C16:0, C18:0, C18:1, C18:2, and C20:0) acylcarnitines (*P* < 0.05) (Figure 1H).

mFAO is critical for generation of ATP and ketone bodies during fasting. The pathway involves repeated cycles of 4 consecutive reactions catalyzed first by acyl-CoA dehydrogenases, then enoyl-CoA hydratases, hydroxyacyl-CoA dehydrogenases, and thiolases, including ACAA2 (5). After each cycle, 2 carbons are cleaved from the acyl-CoA ester to produce an acetyl-CoA molecule; cycles are repeated until FAO is completed. ACAA2 has broad substrate specificity, with the highest thiolase activity for 6–8 carbon fatty-acyl-CoAs. However, only plasma long-chain acylcarnitines were elevated in our patients, especially C20:0 acylcarnitine, which could be a specific biomarker of this syndrome (6).

Inborn deficiencies of most mFAO pathway enzymes follow autosomal recessive inheritance and present with hypoketotic hypoglycemia, cardiomyopathy, myopathy, and hepatic dysfunction (5). Now, we report an autosomal dominant disorder associated with a pathogenic *ACAA2* variant but without serious cardiomyopathy or myopathy. Some patients did have IHH and all adults had FPL and cervical lipomatosis, suggesting an important role of ACAA2 in adipose tissue biology.

We speculate that this variant confers pathological gain of function. ACAA2 also exhibits low acyl-CoA thioesterase activity and can condense acetyl-CoA to form aceto-acetyl-CoA (6). In a reversible reaction, ACAA2 condenses acetyl-CoA with other long-chain acyl-CoAs, which might contribute to elevated plasma long-chain acylcarnitines and excess neutral lipids in hepatocytes. Although excessive accumulation of neutral lipids in adipose tissue can explain cervical lipomatosis, the pathogenesis of lipodystrophy in our patients remains unclear.

## Funding support

This work is the result of NIH funding, in whole or in part, and is subject to the NIH Public Access Policy. Through acceptance of this federal funding, the NIH has been given a right to make the work publicly available in PubMed Central.

- NIH/National Institute of Diabetes and Digestive and Kidney Diseases grant R01-DK105448 (to AG).
- NIH/National Cancer Institute grant R35-CA220449 (to RJD).
- Southwestern Medical Foundation (to AG).
- Intramural Research Program of the National Institute of Diabetes and Digestive and Kidney Diseases (to RJB).
- Cancer Prevention Research Institute of Texas (CPRIT) Core Facilities Support Award RP240494 (to TPM, and RJD).

## Supplementary Material

Supplemental data

Supporting data values

## Figures and Tables

**Figure 1:**
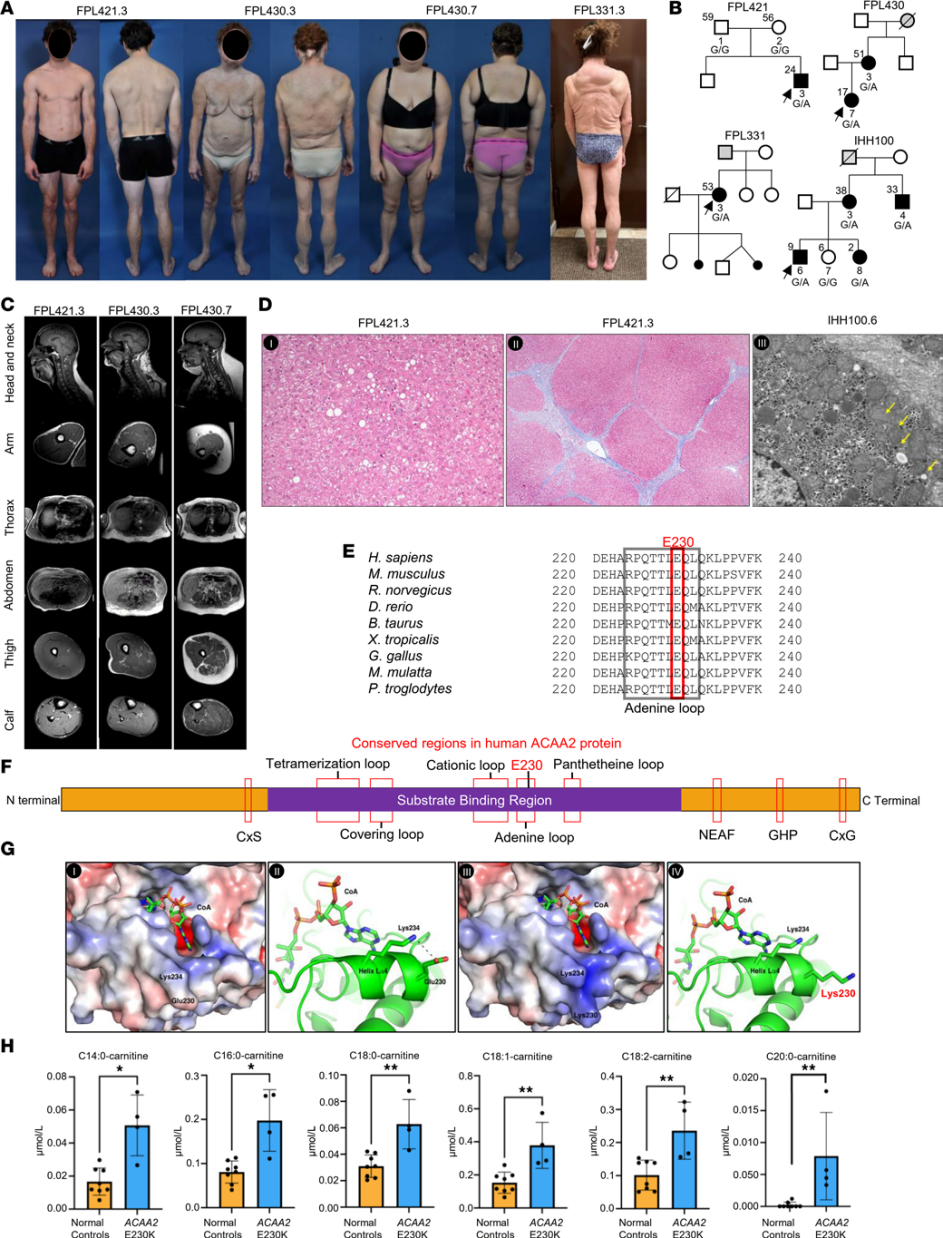
Clinical features, pedigrees, MRI, and liver pathology of patients carrying heterozygous p.Glu230Lys *ACAA2* variant; conservation of Glu230, schematic of ACAA2 structure, effect of variant on ACAA2 structure, and plasma acylcarnitine levels. (**A**) Photographs of 1 male and 3 FPL female patients showing marked loss of extremity fat, especially distally, and lipomatosis in pubic and dorsocervical regions. (**B**) FPL and IHH pedigrees. Black symbols represent affected individuals heterozygous for the c.688G>A (G/A) *ACAA2* variant; white symbols, unaffected individuals (G/G); and gray symbols, possibly having FPL. Age (years) is above symbols. (**C**) T1-weighted MRI of 3 patients with FPL showing variable loss of extremity fat but excess dorsocervical fat. (**D**) Liver histopathology of FPL421.3 (age 18 months) showing micro/macrovesicular steatosis (I) and periportal fibrosis with bridging (II), and of IHH100.6 (age 13 months) (III) showing mitochondria with reduced cristae (yellow arrows) by electron microscopy. Original magnification, ×200 (I), ×40 (II), and ×6,000 (III). (**E**) Multiple species alignment of human ACAA2 showing conservation of Glu230. (**F**) Schematic of human ACAA2 with conserved regions shown in red boxes. (**G**) As compared with wild-type ACAA2 (I, II), the electrostatic potential of the CoA binding surface changes due to variant Glu230Lys (III) (red, negative and blue, positive charge) and it fails to form a salt bridge with Lys234 (IV). (**H**) Plasma long-chain acylcarnitines (median ± SEM) in affected patients (*n* = 4; blue bars) versus normal controls (*n* = 8; orange bars). *P < 0.05, **P < 0.01 (Wilcoxon’s rank sum test).

## Version 1 11/04/2025

In-Press Preview

## Version 2 01/02/2026

Electronic publication
