# Supplementary material for: A recurrent ACAA2 variant causes a dominant syndrome of lipodystrophy, lipomatosis, infantile steatohepatitis, and hypoglycemia
Source: J Clin Invest. 2025 Nov 4;136(1):e198888. doi: 10.1172/JCI198888 (PMC12721882; doi:10.1172/JCI198888)
Supplement: Supplemental data [file jci-136-198888-s014.pdf]

SUPPLEMENTAL MATERIAL

A recurrent *ACAA2* variant causes a dominant syndrome of lipodystrophy, lipomatosis, infantile steatohepatitis and hypoglycemia

Vinaya Simha,[1*] Mary Kate LoPiccolo,[2*] Anna Platt,[3] Rebecca J. Brown,[4] Xandria Johnson,[5] Deanna Alexis Carere,[6] Colleen Donnelly,[2] Matthew T. Snyder,[3] Chao Xing,[5] Thomas P. Mathews,[5] Purva Gopal,[5] Stephen C. Ward,[2] Diana R. Tomchick,[5] Anil K. Agarwal,[5] Ralph J. DeBerardinis,[5] and Abhimanyu Garg[5]

[1]Mayo Clinic, Rochester, Minnesota, USA; [2]Icahn School of Medicine at Mount Sinai, New York, New York, USA; [3]University of Virginia, Charlottesville, Virginia, USA; [4]National Institutes of Health, Bethesda, Maryland, USA; [5]UT Southwestern Medical Center, Dallas, Texas, USA; [6]GeneDx, Gaithersburg, Maryland, USA.

*Shared first co-authors

Address correspondence to: Abhimanyu Garg, 5323 Harry Hines Boulevard, Dallas, Texas 75390-8537. Phone: 1.214.648.2895; email: abhimanyu.garg@utsouthwestern.edu

Funding Support: This work was supported by the National Institutes of Health(NIH)/National Institute of Diabetes and Digestive and Kidney Diseases, R01-DK105448 (to AG), NIH/National Cancer Institute R35-CA220449 (to RJD), Southwestern Medical Foundation (to AG), by the intramural research program of the National Institute of Diabetes and Digestive and Kidney Diseases

(to RJB), and the Cancer Prevention Research Institute of Texas (CPRIT) Core Facilities Support Award RP240494 (to TPM, and RJD). The contributions of the NIH author(s) were made as part of their official duties as NIH federal employees, are in compliance with agency policy requirements, and are considered Works of the United States Government. However, the findings and conclusions presented in this paper are those of the author(s) and do not necessarily reflect the views of the NIH or the U.S. Department of Health and Human Services.

PATIENT PHENOTYPES

All affected adults who were examined (one male and four females) had familial partial lipodystrophy (FPL) with lipomatosis/lipomas in the cervical region and excess fat in the labia majora. During infancy, three had hypoglycemia, with one having severe hypoglycemia-induced brain injury. All of them were managed with frequent feeding during infancy. Six patients had transient hepatitis during infancy. Diabetes mellitus occurred in three patients at ages of 24-49 years. Six patients had hypertriglyceridemia, seven had hepatomegaly, and two had acanthosis nigricans. Two patients had hyperinsulinemia. All patients had low levels of serum leptin and serum alanine aminotransferase or aspartate aminotransferase levels were abnormally high in 4 patients. Details of the phenotypes of individual patients are provided below.

FPL421.3

This 24-year-old white male was born at 37 weeks of gestation with birth weight of 2.5 kg (14th percentile), birth length of 47 cm (21st percentile). He was noticed to have marked loss of subcutaneous fat from the buttocks and legs at birth. He had a viral infection at 3 months, and two months later developed jaundice which resolved after 12 months (Supplemental Figure 4). Liver biopsies revealed features of chronic hepatitis with apoptotic hepatocytes and non-uniform mixed micro/macrovesicular steatosis with mild periportal fibrosis. Electron microscopy revealed increased number of mitochondria, especially in non-steatotic hepatocytes and subtle abnormalities in cristae

and increased mitochondrial pleomorphism in steatotic cells. His serum lactic acid at age of 7.5 months was 5.1 mmol/L (normal range 0.5-2.2 mmol/L). Later at age 10 months and 23 months, serum lactic acid levels were 3.5 and 3.04 mmol/L (normal range <3.33 mmol/L), respectively. At 5 months of age, his serum α-fetoprotein (AFP) level was very elevated at 184,386 ng/mL (normal range 0-9.0 ng/mL). Follow up AFP level at 13 months of age was 13,200 ng/mL and at 28 months of age was 21 ng/mL. It appears that extremely high AFP levels indicate a non-specific inflammatory response during the period of acute hepatitis and the levels normalize after its resolution.

At one year of age, mitochondrial fatty acid oxidation pathway enzyme activities were measured in his skin fibroblasts at the Metabolic Disease Laboratory at the Children's Medical Center at Dallas, Texas, USA (1). The long chain 3-hydroxyacyl-CoA dehydrogenase (LCHAD, also known as mitochondrial trifunction protein, MTP, α subunit) activity for C16 fatty acid in his skin fibroblasts was 107.3 nmol/min/mg protein (normal range 101 ± 22 nmol/min/mg protein) and short-chain 3-hydroxyacyl-CoA dehydrogenase (SCHAD) activity for C4 fatty acid was 173.6 nmol/min/mg protein (normal range 162 ± 38 nmol/min/mg protein). The long-chain 3-keto-acyl CoA thiolase (LCKAT) activity for C16 fatty acid was 71.3 nmol/min/mg protein (normal range 66 ± 13 nmol/min/mg protein) and short chain 3 keto acyl thiolase (also known as β-keto thiolase) activity for C4 fatty acid was 63.9 nmol/min/mg protein (normal range 65 ± 13 nmol/min/mg protein). Palmitic acid (C16:0) oxidation in skin fibroblasts was 46 ± 17 pmol/min/mg protein (normal range 30 ± 5 pmol/min/mg protein) and myristic acid (C14:0) oxidation was 40 ± 10 pmol/min/mg protein (normal range 27 ± 4 pmol/min/mg protein).

His plasma acylcarnitine profile was reported to be normal by a clinical laboratory. At 18 months of age, a liver biopsy sample was sent for acylcarnitine analysis and revealed high levels of acetyl (C2:0) carnitine 24.88 nmol/g wet weight (normal range 5.7 to 23.3 nmol/g wet weight), oleyl

(C18:1) carnitine 1.74 nmol/g wet weight (normal range 0.23 to 1.67 nmol/g wet weight) and linoleyl (C18:2) carnitine 3.21 nmol/g wet weight (normal range 0.34 to 1.80 nmol/g wet weight).

Liver biopsies at age 18 months and 32 months showed continued presence of mild chronic inflammation and a decrease in portal fibrosis. He also had a muscle biopsy at 30 months of age, which showed no histological or ultrastructural abnormalities. Feeding difficulties were also noted, likely secondary to eosinophilic esophagitis and a laryngeal cleft which was surgically repaired at 30 months of age.

At age 24 years, he had marked paucity of subcutaneous fat over the extremities and anterior trunk and acanthosis nigricans in the axillae and groin. Liver and spleen were palpable 6 cm and 3 cm below the costal margin, respectively. Bilateral gynecomastia was noted. He had bilaterally descended normal size testes and normal size phallus. He was taking metformin 500 mg twice daily for diabetes mellitus. An abdominal ultrasound showed hepatosplenomegaly with increased echogenicity. Magnetic resonance elastography showed hepatic steatosis with mean proton density fat-fraction of 17% and liver stiffness at the upper limit of normal (2.4 kPa). His serum testosterone (472 ng/dL, normal 240-950 ng/dL), LH (6.2 IU/L, normal 1.3-9.6 IU/L), TSH (1.8 mIU/L, normal 0.3-4.2 mIU/L) and prolactin levels (10.7 ng/mL, normal 4-15.2 ng/mL) were normal while serum estradiol was slightly elevated at 46 pg/mL (normal: 10-40 pg/mL).

Resting electrocardiogram showed mild repolarization abnormalities (T wave inversions in inferolateral leads) and echocardiogram and cardiac magnetic resonance imaging showed mild concentric left ventricular hypertrophy (posterior wall thickness of 12-13 mm) and borderline abnormal global longitudinal strain (-18%). These changes have been stable after two years follow up and he has good exercise capacity. Coronary computerized tomography scan showed no evidence of subclinical atherosclerosis.

His parents are non-consanguineous and there was no family history of lipodystrophy or metabolic diseases. His mother's body mass index was 20.6 kg/m^2 and the father's body mass index was 26.3 kg/m^2.

FPL430.3

This 51-year-old white female was diagnosed with FPL at 15 years of age. At six months of age, she had "hepatitis" requiring hospital admission for six weeks. She developed diabetes at the age of 35 years and had fatty liver at age 50 years. She had multiple surgeries to resect excess subcutaneous fat from the anterior and posterior neck, shoulders, arms and labia majora starting at the age of 10 years. She had breast reduction at the age of 19 years. She had loss of subcutaneous fat from her cheeks, temporal region, upper and lower extremities and anterior trunk and acanthosis nigricans in the neck, axillae and groin. She had excess fat depots in her lower cheeks, in the dorsal cervical region, around the right clavicle, and in the labia majora. Her liver was palpable 6 cm below the costal margin.

She had a left ovarian cyst removal at 17 years of age and right ovarian dermoid cyst removal at 37 years of age. There was a 2 cm round lipoma on the right antecubital region. Her medications included metformin 1 g twice daily, acyclovir 1 g daily, canagliflozin 100 mg daily, ropinirole 0.5 mg daily, gabapentin 600 mg daily, vitamin D, levothyroxine and semaglutide 2 mg subcutaneously weekly. She had multiple cherry angiomas (0.5 mm to 3 mm) all over the abdomen and thorax.

FPL430.7

This 17-year-old white female presented with muscular legs and a marked loss of subcutaneous fat from the buttocks, hips, legs and forearms. She had increased subcutaneous fat on the upper arms, anterior and posterior neck and posterior thoracic region. She had a birth weight of

4.25 kg. She had menarche at age 12 years and had irregular menstrual periods. At 16 years of age, she developed hypothyroidism and was taking levothyroxine replacement therapy.

FPL331.3

This 53-year-old white female noted increased fat deposition in the submandibular region, upper back, and neck at age 13 years and was diagnosed with FPL in her early 20s. She had liposuction of fat from the upper neck at the age of 17 and 24 years and developed diabetes at age 38 years. At presentation, she had about 14 discrete lipomas in the dorsocervical and sub-occipital region ranging in size from 2 cm to 10 cm diameter and increased fat in the mons pubis and labia majora. She had marked loss of fat from the buttocks, thighs and calves. She had hepatomegaly 5 cm below the costal margin and hepatic steatosis by ultrasonography.

She had irregular menstrual periods since menarche at age 13 years. She developed hypertriglyceridemia at 12 years of age. She had four pregnancies but needed assistance for pregnancies. She has been taking metformin since age 20 years and recently started semaglutide 2 mg subcutaneously weekly. A groin lymph node biopsy at age 48 years revealed fatty infiltration.

IHH100.6

This 9-year-old white male presented at 5 months of age with hepatomegaly, elevated liver enzymes, cholestasis, failure to thrive, and hypoglycemia (plasma glucose 47 mg/dL). Plasma C-peptide level was 2.6 ng/mL (normal 0.7 -9.1 ng/mL). His serum lactic acid level was 4.22 mmol/L (normal range 0.5 -2.2 mmol/L), however, lactic acid was normal on subsequent measures. Liver ultrasound showed a nonspecific, diffusely coarse hyperechoic parenchymal pattern and subcentimeter slightly hyperechoic structures likely involuting hemangiomas. His serum AFP level was very elevated at 184,386 ng/mL (normal range 0-9.0 ng/mL). Plasma acylcarnitines and amino

acids, and urine organic acids were reported to be normal by a clinical laboratory. Frequent feedings with a high-protein, high complex carbohydrate diet were advised by biochemical geneticists. Liver biopsy at 13 months of age showed patchy hepatocellular swelling and microvesicular steatosis, mild portal and lobular inflammation with portal bridging fibrosis (stage 3-4 fibrosis). Electron microscopy images revealed large numbers of mitochondria with sparse/stunted cristae in some hepatocytes. By 12 months of age, his liver function tests, and fasting tolerance had improved. The patient's AFP level was improving (20,488 ng/mL). At 4 years of age, a lipoma was noted on the back of his neck which continued to grow. Liver fibroscan showed resolution of fibrosis and no steatosis at that time. Serum AFP level was normal. At 8 years of age, the patient had a normal liver ultrasound and echocardiogram. He had central adiposity.

IHH100.8

This 19-month-old female was born at 36 weeks of gestation and stayed in the neonatal intensive care unit for 1 week due to hyperbilirubinemia and hypoglycemia, both attributed to prematurity. She presented at 4 months of age with elevated liver enzymes and direct hyperbilirubinemia. Her serum AFP level was 570,232 ng/mL Given low home capillary blood glucose measurements (48-54 mg/dL) and family history of infantile hypoglycemia, she was fed frequently. Liver ultrasound showed nonspecific, diffusely coarse hyperechoic hepatic parenchymal pattern. Her fasting interval was able to be increased at 8 months of age and serum liver enzymes also improved. At 15 months of age, her serum AFP had decreased markedly, but it remained elevated (3,676 ng/mL). Liver ultrasound and echocardiogram were normal. No abnormal fat distribution has been noted.

IHH100.3

This 38-year-old white female had failure to thrive, hepatomegaly, and jaundice at 5 months of age which resolved spontaneously. She noticed a lipoma on the back of her neck at around 15 years of age which grew markedly during pregnancy such that it encompassed her anterior neck. Her father had similar lipomas in his 40’s, which recurred despite surgical removal. She had increased subcutaneous fat on the upper arms and central adiposity but lack of fat on the lower extremities. Her serum lactic acid was normal (0.74 mmol/L) at 38 years of age. She had a normal echocardiogram and no limitation in exercise capacity.

IHH100.4

This 33-year-old white male had severe intellectual disability and a seizure disorder secondary to severe hypoglycemia-induced brain injury at 5 months of age. He also had a history of jaundice, hepatomegaly, cirrhosis, and failure to thrive. He was treated as if he had long-chain 3-hydroxyacyl-coenzyme A dehydrogenase (LCHAD) deficiency until adolescence, as that was the presumed diagnosis. This involved a low-fat diet with medium chain triglyceride (MCT) supplementation. The hypoglycemia resolved spontaneously with frequent feeding and fasting avoidance. He is non-ambulatory, non-verbal, and has a gastrostomy-tube. He does not have obvious lipomatosis but has muscle wasting. His echocardiogram was normal. His plasma acylcarnitine profile was reported to be normal by a clinical laboratory.

RESEARCH DESIGN AND METHODS

Study Approvals: Study protocols were approved by the Institutional Review Boards of Mayo Clinic, Rochester, Minnesota; Icahn School of Medicine at Mount Sinai, New York, New York; UT Southwestern Medical Center, Dallas, Texas; and the National Institutes of Health (NIH), Bethesda, Maryland, USA. All affected patients from pedigrees FPL421, FPL430 and FPL331, and healthy controls provided written informed consent at UT Southwestern. In addition, the patient FPL421.3 provided written informed consent at the Mayo Clinic and the patient FPL331.3 provided written informed consent at the NIH. Written informed consent was received for the use of the photographs and the record of informed consent has been retained. For pedigree IHH, the request for a waiver of informed consent was approved by the IRB of Icahn School of Medicine at Mount Sinai, New York, New York.

Sex as a biological variable: Lipodystrophy patients and their family members were ascertained regardless of sex.

Anthropometry: Skinfold thickness was measured with a Lange caliper on the right side of the body.(2) Height and body weight were measured with standard procedures.

Dual Energy X-Ray Absorptiometry (DXA) Scan: One male and three females with FPL had whole body DXA scan for total and regional body fat determination (2).

Whole Body Magnetic Resonance Imaging (MRI): MRI studies were performed in a male and two females with FPL using a 3.0 Tesla imaging device (Philips Achieva, usa.Philips.com) as described previously (3).

Molecular Sequencing and Analysis: All probands, and other affected and unaffected relatives underwent whole exome sequencing (WES). For pedigrees, FPL421, FPL430 and IHH100, genomic DNA was isolated from buccal swabs and whole exome sequencing (WES) was performed at GeneDx. Using genomic DNA from the proband, parent(s) and other relatives, the exonic regions and flanking splice junctions of the genome were captured using the Clinical Research Exome (Agilent Technologies, Santa Clara, CA) or the IDT xGen Exome Research Panel v1.0 (Integrated DNA Technologies, Coralville, IA) kit. Massively parallel (NextGen) sequencing was done on an Illumina system with 150bp paired-end reads. Reads were aligned to human genome build GRCh37/UCSC hg19 and analyzed for sequence variants using a custom-developed analysis tool. Reported variants were confirmed, if necessary, by an appropriate orthogonal method in the proband and, if submitted, in selected relatives. Additional sequencing technology and variant interpretation protocol has been previously described (4). The general assertion criteria for variant classification are publicly available on the GeneDx ClinVar submission page (http://www.ncbi.nlm.nih.gov/clinvar/submitters/26957/).

For Patient FPL 430.3, the mean depth of coverage was 135x with 98.3% of bases covered by at least 10 reads (10x). For patient, FPL331.3, genomic DNA was isolated from peripheral blood using the Easy-DNA kit (Invitrogen, Carlsbad, CA) and WES was performed using the Integrated DNA Technologies xGen Exome Research Panel V.1.0 on the Illumina platform. The mean coverage of the targeted regions was 140-fold with 97% of bases covered by >50-fold reads. Sequences were aligned to the human reference genome b37, and variants were called using the Genome Analysis Toolkit (v3.8) and annotated using SnpEff (v5.1). The following genes were specifically reviewed, all 100% of the coding nearby untranslated regions covered with more than 10x: *ADRA2A*, *AKT2*, *CAV1*, *CIDEC*, *LIPE*, *LMNA*, *NOTCH3, PCYT1A*, *PIK3R1*, *PLIN1*, *PPARG*, and *PSMB8*. No reportable variants were identified in the coding regions of these genes covered by this test.

Plasma Acylcarnitine Profile: Plasma from four affected patients, FPL421.3, 430.7, 430.3 and 331.3 and eight control subjects was submitted for acylcarnitine profiling with the Children's Research Institute Metabolomics Facility at UT Southwestern Medical Center, Dallas, Texas. Blood samples for acylcarnitine profile were obtained during the patients' current evaluation and they did not have any hepatitis or decompensation.

Acylcarnitines were extracted and analyzed from human plasma samples as published previously (5-7). Aliquots of 10 µL of plasma were extracted in an ice-cold mixture of 80/20 acetonitrile and water. Ten µL of a 100-fold dilution of the Carnitine/Acylcarnitine Standard Mix Set B (Cambridge Isotope Laboratories, Item No. NSK-B-1) was spiked into the mixture. Extracts were placed into a refrigerated benchtop centrifuge and spun at 17,000xg for 10 minutes. The supernatant was removed and placed into LC/MS vials for analysis. Data were acquired on a Thermo Scientific (Bremen, Germany) Vanquish Flex liquid chromatography system using HILIC chromatography coupled to an Orbitrap Exploris 480 mass spectrometer.

Statistics: Plasma acylcarnitines levels were compared by the Wilcoxon rank sum test implemented in R package coin.

EXTENDED RESULTS

The variant, c.688G>A, p.Glu230Lys, in *ACAA2* had a GERP++ score of 5.79 and CADD score of 28.3 consistent with it being 'probably damaging'. The variant was not present in GnomAD (https://gnomad.broadinstitute.org), UK Biobank (https://www.ukbiobank.ac.uk), or All of Us (https://allofus.nih.gov). Single cell data suggest that the ACAA2 transcript is in skin fibroblasts (https://www.gtexportal.org/home/gene/ACAA2). Also, protein expression has been detected in adipose tissue fibroblasts (https://www.proteinatlas.org/ENSG00000167315-ACAA2/single+cell/adipose+tissue#tissue_cell_type).

Analysis of the Effects of Glu230Lys Variant on ACAA2 Structure: ACAA2 variant was modeled using ACAA2 x-ray structure (accession number: PDB ID 4C2J) employing a widely available PyMOL software (8). The crystal structure reported for human ACAA2 bound to Coenzyme A (CoA) is at a resolution of 2.0 Å (8). The ACAA2 structure is tetrameric (9-11). The monomer of human ACAA2 adopts the canonical thiolase fold, an α/β fold of two subdomains, wherein the amino-terminal residues 1-126 and 254-272 form a mixed β-sheet sandwiched between α-helices in an alternating "βα" pattern βα βα βα ββ, while the carboxy-terminal residues 273-396 form a four-stranded mixed β-sheet. Dimerization occurs through the Nβ3 strands (residues 85-89) of each monomer. Residues 127-253 form a loop domain that contains 4 helices and folds on top of the thiolase core domain. The loop domain is essential for enzyme activity in both the degradative and synthetic directions as well as for the acyl-CoA thioesterase activity, as it is involved in fatty acyl-CoA and CoA binding and contains structural features that determine substrate specificity. Residues 127-146 of the loop domain from all four monomers mediate the tetramerization of the enzyme.

Although Kiema et al.(8) co-crystallized the enzyme with octanoyl-CoA, low levels of acyl-CoA thioesterase activity of ACAA2 resulted in a structure with one bound molecule of CoA per monomer. The catalytic residues are in a deep pocket between the N- and C-terminal subdomains of each monomer, with Cys92 located in the amino-terminal portion of the protein, while Asn320, His352, and Cys382 are in the carboxy-terminus of the protein. The molecule of CoA binds with the cysteamine sulfur of the coenzyme within 5 Å of the catalytic cysteines. A tunnel that extends into the adjacent monomer of the dimer is the putative binding site for the fatty-acyl tail of fatty-acyl-CoA substrates, which would place the acyl-CoA moiety near the catalytic Cys92. While the precise enzymatic mechanism is not well known for these types of thiolases, the region of the protein that includes residue Glu230 is presumed to adopt different conformations during catalysis.

This orientation of the CoA moiety binding is similar to that seen in other thiolases such as the *Saccharomyces cerevisiae* peroxisomal 3-ketoacyl-CoA thiolase (12). Residues His222-Val253 of the loop domain of ACAA2 are specifically involved in the binding of the CoA moiety for all substrates.(8) This region of the protein forms a positively charged groove that sandwiches the adenine base and part of the ribose ring of the CoA. The residue Glu230 is near the N-terminal end of helix Lα4 (residues 228-235), which forms part of this positively charged groove. The presence of the positively charged CoA binding groove may be an example of electrostatic preorganization that allows the wild type enzyme to correctly position the substrate within the active site (13), and the increase in positive charge near the CoA binding groove in the E230K mutant may enhance this effect.

Glu230 and Lys234 are located on the outer surface of ACAA2 and form a salt bridge. The substitution of Glu230 to Lys results in increased positive charge of the amino-terminus of helix Lα4 and a loss of this crucial salt bridge. These structural changes in ACAA2 are predicted to alter the K_m for the substrate(s) through electrostatic interactions with the negatively charged 3'-phosphoadenosine of the CoA portion of 3-ketoacyl-CoA. These changes may enhance the binding of CoA to the active site in the enzyme. However, we do not have direct ACAA2 enzyme activity data.

EXTENDED DISCUSSION

Inborn deficiencies of most enzymes of the mFAO pathway have been well described and follow an autosomal recessive inheritance pattern.(14, 15) These disorders usually present with elevated risk of life-threatening 'hypoketotic hypoglycemia' especially during catabolic conditions such as fasting, exercise, and illness. Other common clinical features of the mFAO disorders include cardiomyopathy with arrhythmias and conduction defects, myopathy with rhabdomyolysis and hepatic dysfunction.(14) Although MCKAT deficiency has been reported in a Japanese neonate who died of metabolic acidosis, liver dysfunction and rhabdomyolysis, based on absent enzymatic activity in cultured fibroblasts,(16) a causal variant in *ACAA2* was not been reported in this patient.

Interestingly, despite reportedly normal plasma acylcarnitine levels by clinical laboratories in four of our affected patients, we found significant elevations in the plasma levels of long chain-acylcarnitines in our patients. Some of these clinical laboratories did not measure the levels of plasma C20:0 acylcarnitine.

Usually, deficiencies of the enzymes involved in the mFAO pathway result in accumulation of specific plasma acylcarnitines in plasma corresponding to the fatty acids these enzymes prefer to oxidize. For example, in SCAD and SCHAD deficiencies, C4:0 and C4-OH acylcarnitines accumulate in plasma, respectively; in MCAD deficiency, C6:0, C8:0 and C10:1 acylcarnitines; in LCHAD and LCKAT deficiencies, C14-OH, C16-OH, C18:0-OH and C18:1-OH acylcarnitines; and in VLCAD deficiency, C14:1 acylcarnitine accumulates.(15) Although ACAA2 prefers six to eight carbon fatty-acyl-CoAs,(8) our patients did not have high plasma levels of C6:0 or C8:0 acylcarnitines. Furthermore, documentation of normal mFAO pathway enzyme activities and oxidation of palmitic and myristic acid in skin fibroblasts of one patient make it unlikely that this is a complete loss-of-function variant. Therefore, we suggest that the variant could be associated with a gain of function. The disease is associated with a recurrent, monoallelic variant. We don't know of other patients with this disease who have different variants in ACAA2. That makes haploinsufficiency less likely, and besides, we are not aware of any severe phenotypes associated with haploinsufficiency of any mFAO enzyme. The effects of the variant on ACAA2 structure also suggest that it may enhance the binding of Coenzyme A to the active site in the enzyme. Future investigations will be required to understand how this variant alters various different enzyme activities of ACAA2 and results in elevations of plasma long chain acylcarnitines. In mFAO pathway defects, hypoglycemia arises during prolonged fasts because the lack of ketone bodies deprives tissues of a crucial alternative fuel.(14, 15) We speculate that the causative ACAA2 variant reported here induces secondary inhibition of mFAO pathways, and thus can induce hypoglycemia in our patients under stressful situations.

Early recognition of this genetic disorder can prevent serious and life-threatening hypoglycemia and its sequelae and possibly steatohepatitis during infancy. Whether any specific dietary modifications or other novel therapies targeting mFAO pathways will improve morbidity in patients with this *ACAA2* variant remains to be studied. Marked hypoleptinemia suggests a role for leptin replacement therapy in improving metabolic complications of FPL in our patients.

Data availability: The WES datasets cannot be released by the Health Insurance Portability and Accountability Act (HIPAA) and the Genetic Information Nondiscrimination Act (GINA) to protect patients' genetic privacy. The individual plasma acylcarnitine levels can be requested from the corresponding author. Values for all data points in graphs are reported in the Supporting Data Values file.

Acknowledgments: The authors thank Helen Hobbs, M.D., UT Southwestern, for helpful discussions during preparation for this manuscript; Tea Huseinbegovic, B.S., Mary Tunison, M.S., Alec Whited, M.S., UT Southwestern for illustrations; Claudia Quittner, M.S., UT Southwestern for patient care; Xilong Li, Ph.D. from the Peter O'Donnell Jr. School of Public Health, UT Southwestern Medical Center, Dallas, Texas for analysis of DXA data; and GeneDx for conducting WES and arranging communication between various investigators. We acknowledge the Texas Advanced Computing Center (https://www.tacc.utexas.edu) at the University of Texas at Austin for providing high performance computing resources that have contributed to the research results reported within this paper. This research was supported in part by the Intramural Research Program of the National Institutes of Health (NIH). The contributions of the NIH author(s) were made as part of their official duties as NIH federal employees, are in compliance with agency policy requirements, and are considered Works of the United States Government. However, the findings and conclusions

presented in this paper are those of the author(s) and do not necessarily reflect the views of the NIH or the U.S. Department of Health and Human Services.

Author contributions: VS, MKL, CX, CD, AP, MTS, RJB and AG conceived the research, designed, conducted and supervised experiments, acquired and interpretated data and wrote the manuscript; DAC and CX analyzed whole exome sequencing data; VS, MKL, RJB, AG conducted in-depth phenotyping of the patients and edited the manuscript; XJ, TPM and RJD conducted metabolomics studies on the plasma samples and interpreted the data; AKA and DRT conducted analysis of the variant on ACAA2 structure; PG and SW reviewed liver biopsy slides and electron microscopic images. All authors reviewed and edited the manuscript. VS identified the first pedigree with ACAA2 variant and MKL identified the last pedigree and therefore, VS is listed as first author followed by MKL.

Financial Disclosures: AG and RJB received research support from Chiesi and Regeneron. DAC is a paid employee of GeneDx and owns GeneDx stock. RJD has received consulting fees from Atavistik Bioscience, Faeth Therapeutics, General Metabolics and Vida Ventures. All other authors have no conflict of interest.

Supplemental Table 1. Salient clinical characteristics of Familial Partial Lipodystrophy related to different genetic causes

Clinical features	FPL Subtypes (Gene)(References)									
	Autosomal Dominant							Autosomal Recessive		
	**(*ACAA2*)**	**FPLD2 (*LMNA*)** (17-19)	**FPLD3 (*PPARG*)** (19, 20)	**(*AKT2*)** (21)	**FPLD4 (*PLIN1*)** (22-25)	**FPLD1 (*NOTCH3*)** (26)	**FPLD8 (*ADRA2A*)** (27)	**FPLD5 (*CIDEC*)** (28)	**FPLD6 (*LIPE*)** (29-33)	**(*PCYT1A*)** (34)
Pedigrees (n)	4	>400	>50	1	10	3	1	1	7	2
Patients (n)	8	>680	>100	4	23	6	3	1	13	2
Onset of fat loss	Early childhood	Childhood	1st- 2nd decade	NR	1st-2nd decade	1st-2nd decade	Post puberty	Early childhood	Adult	Early childhood
Areas of predominant fat loss	Extremities, variable amounts from trunk, mainly anterior	Extremities, variable amounts from trunk, mainly anterior	Extremities, but not very striking	Gluteal region	Extremities	Extremities, hips	Extremities, trunk	Extremities	Lower extremities	Lower extremities, hips
Areas of excess fat deposition	Upper back	Face, neck, upper back	Face	NR	Face, upper back	NR	Face, upper back	NR	Face, back	NR
Additional lipomas or lipomatosis	Over upper back in some patients	Rare lipomas	NR	NR	NR	NR	NR	NR	Multiple symmetric lipomatosis	NR
Prominent muscularity	Yes	Yes	Yes	No	Yes	Yes	Yes	Yes	Yes	No
Metabolic complications	DM, HTG, MASLD	DM, HTG, MASLD, PCOS	Severe for extent of fat loss	DM	DM, HTG, MASLD	Early DM, HTG, MASLD	DM, HTG, MASLD	DM with DKA, HTG	Mild	DM, HTG, MASLD
Other associated conditions	Infantile hepatitis/ hypoglycemia	Cardiomyopathy, Myopathy, Cushingoid appearance			Acromegaloid, Cushingoid appearance	No excess fat in labia, Glomus tumors	Heat intolerance, hot flashes	Multilocular adipocytes on histology	Distal symmetric myopathy, High CK level	Short stature

Abbreviations: CK, creatine phosphokinase; DKA, diabetic ketoacidosis; DM, diabetes mellitus; HTG, hypertriglyceridemia; MASLD, metabolic dysfunction associated steatotic liver disease; NR, not reported; PCOS, polycystic ovarian syndrome;

Supplemental Table 2. Clinical characteristics and metabolic variables of our patients with the *ACAA2* variant

	FPL 421.3	FPL 430.3	FPL 430.7	FPL 331.3	IHH 100.3	IHH 100.4	IHH 100.6	IHH 100.8
Age (years)	24	51	17	53	38	33	8	1.6
Sex	M	F	F	F	F	M	M	F
Height (m)	1.89	1.64	1.60	1.61	1.60	1.42	1.32	0.79
Weight (kg)	86.9	65.1	77.2	66.3	65.3	48.9	32.5	12.0
BMI (kg/m^2)	24.0	24.2	30.2	25.6	25.5	24.2	18.6	19.5
Diabetes (onset age, years)	+ (24)	+ (35)	-	+ (49)	-	-	-	-
Hypertriglyceridemia	+	+	+	+	-	+	+	NA
Acanthosis nigricans	+	+	-	-	-	-	-	-
Hypoglycemia (age, months)	-	-	-	-	-	+ (5)	+ (5)	+ (4)
Steatohepatitis (age, months)	+ (5)	+ (5)	-	-	+ (5)	+ (5)	+ (5)	+ (4)
Hepatomegaly	+	+	-	+	+	+	+	+
Partial Lipodystrophy	+	+	+	+	+	NA	-	-
Lipomatosis/ Lipomas	-	+	+	+	+	NA	+	NA
Upper Extremity fat† (%)	13.8	20.5	40.3	22.7	NA	NA	NA	NA
Lower Extremity fat† (%)	7.2	9.8	21.7	14.7	NA	NA	NA	NA
Trunk fat† (%)	8.0	17.7	26.6	16.2	NA	NA	NA	NA
Head fat† (%)	NA	19.1	20.1	26.6	NA	NA	NA	NA
Total fat† (%)	9.3	16.1	26.7	17.5	NA	NA	NA	NA
Glucose (mg/dL)	102	177	94	162	87	84	88	NA
Insulin (μU/mL)	75.6	15.8	16.4	38.6	NA	NA	NA	NA
Triglycerides (mg/dL)	333	160	276	249	93	210	221	NA

Cholesterol (mg/dL)	189	160	136	111	197	139	245	NA
HDL-C (mg/dL)	41	50	38	27	51	23	16	NA
HbA1c (%)	6.2	7.7	5.2	7.4	NA	NA	NA	NA
Leptin (ng/mL)	0.3	1.6	2.7	1.6	NA	NA	NA	NA
AST (U/L)	35	44	31	14	27	67	38	NA
ALT (U/L)	59	87	55	9	28	76	35	NA

Abbreviations: BMI, body mass index; M, male; F, female; †Assessed using whole body Dual Energy X-ray Absorptiometry. AST, aspartate aminotransferase; ALT, alanine aminotransferase; HbA1c, hemoglobin A1c; HDL-C, high density lipoprotein cholesterol. All values are for fasting samples. NA, not available. Normal values of biochemical variables are as follows: Glucose, <100 mg/dL; Insulin ≤ 18.4 µU/mL; Triglycerides, < 150 mg/dL; Cholesterol, < 200 mg/dL; HDL-C, males ≥ 40 mg/dL; Females ≥ 50 mg/dL; HbA1c, 4.0 -5.6%; Leptin, Males 0.3-19.9 ng/mL; Females, 4.7-38.9 ng/mL; AST 13-39 U/L; ALT 7-52 U/L.

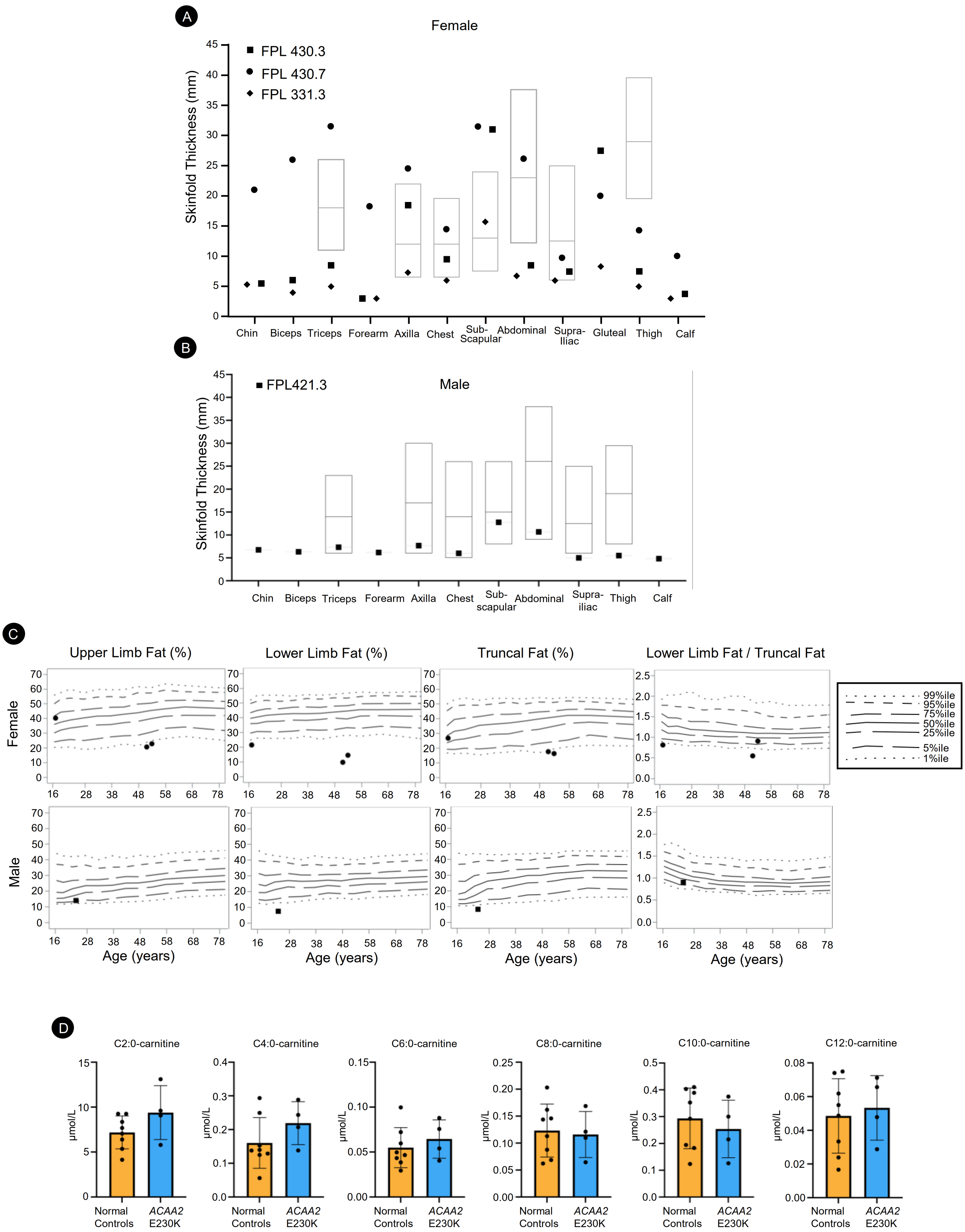
A
Female
FPL 430.3
FPL 430.7
FPL 331.3
Skinfold Thickness (mm)
Chin
Biceps
Triceps
Forearm
Axilla
Chest
Sub-Scapular
Abdominal
Supra-Iliac
Gluteal
Thigh
Calf
B
FPL421.3
Male
Skinfold Thickness (mm)
Chin
Biceps
Triceps
Forearm
Axilla
Chest
Sub-scapular
Abdominal
Supra-iliac
Thigh
Calf
C
Upper Limb Fat (%)
Lower Limb Fat (%)
Truncal Fat (%)
Lower Limb Fat / Truncal Fat
Female
Male
99%ile
95%ile
75%ile
50%ile
25%ile
5%ile
1%ile
Age (years)
D
C2:0-carnitine
C4:0-carnitine
C6:0-carnitine
C8:0-carnitine
C10:0-carnitine
C12:0-carnitine
μmol/L
Normal Controls
ACAA2 E230K

Supplemental Figure 1. Skinfold thickness, regional body fat and plasma short and medium chain acyl-carnitines of affected patients and controls. (A) Skinfold thickness measurements of three affected females (FPL430.3, FPL430.7 and FPL331.3) with heterozygous c.688G>A *ACAA2* variant. Each symbol represents a patient. The bars represent 10th and 90th percentile values of normal women with horizontal line in the middle of the bars representing the median value (35). (**B**) Skinfold thickness measurements of the affected male patient (FPL421.3) with heterozygous c.688G>A *ACAA2* variant. The bars represent 10th and 90th percentile values of normal men with horizontal line in the middle of the bars representing the median value (36). (**C**) Comparison of regional body fat by dual-energy x-ray absorptiometry (DXA) scan of FPL patients with *ACAA2* variant with the National Health and Nutrition Examination Survey (NHANES) controls. The data from the NHANES controls have been drawn as sex- and age-specific percentile values for comparison. The proportion of fat in specific body regions as well as whole body was calculated as a percentage of body mass (2, 37). Mean values of the right and left upper limb fat and right and left lower limb fat (% of regional fat) were calculated. Data from the sex- and age-matched controls from the NHANES from 4 survey cycles, 1999-2000, 2001-2002, 2003-2004, and 2005-2006 for DXA data were used for comparison. The NHANES percentiles was calculated using SAS 9.4 (SAS Institute, Cary, NC). Two of the three affected females had upper limb fat ≤1st percentile of NHANES. All affected females had lower limb fat ≤1st percentile of NHANES. Truncal fat was ≤1st percentile of NHANES in two affected females. Lower limb fat/truncal fat ratio was ≤1st percentile of NHANES in two affected females. The affected male had upper limb fat ~5th percentile, lower limb fat ≤1st percentile, truncal fat <1st percentile and the ratio of lower limb fat/truncal fat was ~10th percentile of NHANES. (**D)** Plasma short- and medium-chain acylcarnitine levels (median and SEM) in affected patients (n=4; blue bars) and in normal controls (n=8; orange bars). There was no statistical difference in the levels of the two groups by Wilcoxon rank sum test.

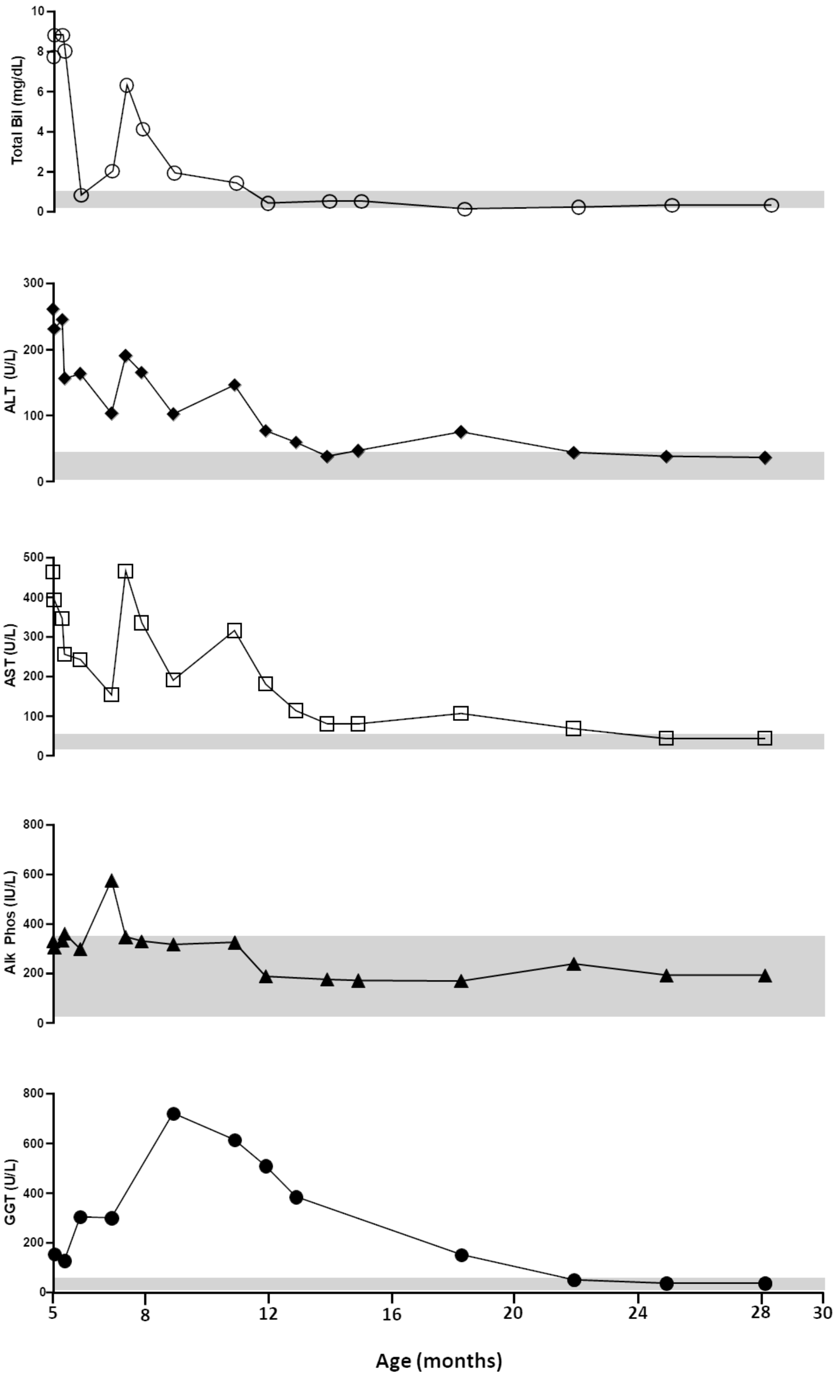

Supplemental Figure 2. Liver function tests of the patient FPL421.3 during infancy and early childhood. He developed jaundice at 5 months of age and most of the liver function tests normalized spontaneously by 1.5 years of age without any specific treatment. The gray regions indicate normal range for each variable. Normal ranges are as follows: Total Bilirubin, 0.2 to 1.0 mg/dL; AST, 15-54 U/L; ALT, 3-44 U/L; Alk Phos, 25-346 IU/L; and GGT, 8-55 U/L. Abbreviations: Bil, bilirubin; AST, aspartate transaminase; ALT, alanine transaminase; Alk Phos, alkaline phosphatase; GGT, gamma-glutamyl transferase.

References:

1. Bennett MJ, Sheng F, and Saada A. Biochemical assays of TCA cycle and beta-oxidation metabolites. *Methods Cell Biol.* 2020;155:83-120.
2. Vasandani C, Li X, Sekizkardes H, Adams-Huet B, Brown RJ, and Garg A. Diagnostic Value of Anthropometric Measurements for Familial Partial Lipodystrophy, Dunnigan Variety. *J Clin Endocrinol Metab.* 2020;105(7):2132-41.
3. Garg A, Peshock RM, and Fleckenstein JL. Adipose tissue distribution pattern in patients with familial partial lipodystrophy (Dunnigan variety). *J Clin Endocrinol Metab.* 1999;84(1):170-4.
4. Retterer K, Juusola J, Cho MT, Vitazka P, Millan F, Gibellini F, et al. Clinical application of whole-exome sequencing across clinical indications. *Genet Med.* 2016;18(7):696-704.
5. Aurora AB, Khivansara V, Leach A, Gill JG, Martin-Sandoval M, Yang C, et al. Loss of glucose 6-phosphate dehydrogenase function increases oxidative stress and glutaminolysis in metastasizing melanoma cells. *Proc Natl Acad Sci U S A.* 2022;119(6).
6. DeVilbiss AW, Zhao Z, Martin-Sandoval MS, Ubellacker JM, Tasdogan A, Agathocleous M, et al. Metabolomic profiling of rare cell populations isolated by flow cytometry from tissues. *Elife.* 2021;10.
7. Pachnis P, Wu Z, Faubert B, Tasdogan A, Gu W, Shelton S, et al. In vivo isotope tracing reveals a requirement for the electron transport chain in glucose and glutamine metabolism by tumors. *Sci Adv.* 2022;8(35):eabn9550.
8. Kiema TR, Harijan RK, Strozyk M, Fukao T, Alexson SE, and Wierenga RK. The crystal structure of human mitochondrial 3-ketoacyl-CoA thiolase (T1): insight into the reaction mechanism of its thiolase and thioesterase activities. *Acta Crystallogr D Biol Crystallogr.* 2014;70(Pt 12):3212-25.
9. Staack H, Binstock JF, and Schulz H. Purification and properties of a pig heart thiolase with broad chain length specificity and comparison of thiolases from pig heart and Escherichia coli. *J Biol Chem.* 1978;253(6):1827-31.
10. Uchida Y, Izai K, Orii T, and Hashimoto T. Novel fatty acid beta-oxidation enzymes in rat liver mitochondria. II. Purification and properties of enoyl-coenzyme A (CoA) hydratase/3-hydroxyacyl-CoA dehydrogenase/3-ketoacyl-CoA thiolase trifunctional protein. *J Biol Chem.* 1992;267(2):1034-41.
11. Miyazawa S, Furuta S, Osumi T, Hashimoto T, and Ui N. Properties of peroxisomal 3-ketoacyl-coA thiolase from rat liver. *J Biochem.* 1981;90(2):511-9.
12. Mathieu M, Modis Y, Zeelen JP, Engel CK, Abagyan RA, Ahlberg A, et al. The 1.8 A crystal structure of the dimeric peroxisomal 3-ketoacyl-CoA thiolase of Saccharomyces cerevisiae: implications for substrate binding and reaction mechanism. *J Mol Biol.* 1997;273(3):714-28.
13. Fried SD, and Boxer SG. Electric Fields and Enzyme Catalysis. *Annu Rev Biochem.* 2017;86:387-415.
14. Houten SM, Violante S, Ventura FV, and Wanders RJ. The Biochemistry and Physiology of Mitochondrial Fatty Acid beta-Oxidation and Its Genetic Disorders. *Annu Rev Physiol.* 2016;78:23-44.
15. Vianey-Saban C, Guffon N, Fouilhoux A, and Acquaviva C. Fifty years of research on mitochondrial fatty acid oxidation disorders: The remaining challenges. *J Inherit Metab Dis.* 2023;46(5):848-73.
16. Kamijo T, Indo Y, Souri M, Aoyama T, Hara T, Yamamoto S, et al. Medium chain 3-ketoacyl-coenzyme A thiolase deficiency: a new disorder of mitochondrial fatty acid beta-oxidation. *Pediatr Res.* 1997;42(5):569-76.
17. Besci O, Foss de Freitas MC, Guidorizzi NR, Guler MC, Gilio D, Maung JN, et al. Deciphering the Clinical Presentations in LMNA-related Lipodystrophy: Report of 115 Cases and a Systematic Review. *J Clin Endocrinol Metab.* 2024;109(3):e1204-e24.

18. Anum, Li X, and Garg A. Novel and Ultra-Rare Heterozygous Mis-sense LMNA variants causing Familial Partial Lipodystrophy. *J Clin Endocrinol Metab.* 2025.
19. Vasandani C, Li X, Sekizkardes H, Brown RJ, and Garg A. Phenotypic Differences Among Familial Partial Lipodystrophy Due to LMNA or PPARG Variants. *J Endocr Soc.* 2022;6(12):bvac155.
20. Hussain I, Patni N, and Garg A. Lipodystrophies, dyslipidaemias and atherosclerotic cardiovascular disease. *Pathology.* 2019;51(2):202-12.
21. George S, Rochford JJ, Wolfrum C, Gray SL, Schinner S, Wilson JC, et al. A family with severe insulin resistance and diabetes due to a mutation in AKT2. *Science.* 2004;304(5675):1325-8.
22. Gandotra S, Le Dour C, Bottomley W, Cervera P, Giral P, Reznik Y, et al. Perilipin deficiency and autosomal dominant partial lipodystrophy. *N Engl J Med.* 2011;364(8):740-8.
23. Jeru I, Vantyghem MC, Bismuth E, Cervera P, Barraud S, Group PL-S, et al. Diagnostic Challenge in PLIN1-Associated Familial Partial Lipodystrophy. *J Clin Endocrinol Metab.* 2019;104(12):6025-32.
24. Kozusko K, Tsang V, Bottomley W, Cho YH, Gandotra S, Mimmack ML, et al. Clinical and molecular characterization of a novel PLIN1 frameshift mutation identified in patients with familial partial lipodystrophy. *Diabetes.* 2015;64(1):299-310.
25. Chen X, and Goodman JM. The collaborative work of droplet assembly. *Biochim Biophys Acta Mol Cell Biol Lipids.* 2017;1862(10 Pt B):1205-11.
26. Garg A, Xing C, Agarwal AK, Westfall AK, Tomchick DR, Zhang X, et al. Gain of Function NOTCH3 Variants Cause Familial Partial Lipodystrophy Due to Activation of Senescence Pathways. *Diabetes.* 2025;74(3):427-38.
27. Garg A, Sankella S, Xing C, and Agarwal AK. Whole-exome sequencing identifies ADRA2A mutation in atypical familial partial lipodystrophy. *JCI Insight.* 2016;1(9).
28. Rubio-Cabezas O, Puri V, Murano I, Saudek V, Semple RK, Dash S, et al. Partial lipodystrophy and insulin resistant diabetes in a patient with a homozygous nonsense mutation in CIDEC. *EMBO Mol Med.* 2009;1(5):280-7.
29. Mohan V, Damle VA, Patil AV, Lavanya R, Vijayalakshmi K, Regina A, et al. Interesting Case of Familial Partial Lipodystrophy Syndrome (Type 6) with LIPE Gene Defect: A Case Report. *J Assoc Physicians India.* 2025;73(5):93-4.
30. Zhou Y, Zhang L, Ding Y, and Zhai Y. Case report: First Chinese patient with family partial lipodystrophy type 6 due to novel compound heterozygous mutations in the LIPE gene. *Front Genet.* 2024;15:1417613.
31. Sollier C, Capel E, Aguilhon C, Smirnov V, Auclair M, Douillard C, et al. LIPE-related lipodystrophic syndrome: clinical features and disease modeling using adipose stem cells. *Eur J Endocrinol.* 2021;184(1):155-68.
32. Zolotov S, Xing C, Mahamid R, Shalata A, Sheikh-Ahmad M, and Garg A. Homozygous LIPE mutation in siblings with multiple symmetric lipomatosis, partial lipodystrophy, and myopathy. *Am J Med Genet A.* 2017;173(1):190-4.
33. Farhan SM, Robinson JF, McIntyre AD, Marrosu MG, Ticca AF, Loddo S, et al. A novel LIPE nonsense mutation found using exome sequencing in siblings with late-onset familial partial lipodystrophy. *Can J Cardiol.* 2014;30(12):1649-54.
34. Payne F, Lim K, Girousse A, Brown RJ, Kory N, Robbins A, et al. Mutations disrupting the Kennedy phosphatidylcholine pathway in humans with congenital lipodystrophy and fatty liver disease. *Proc Natl Acad Sci U S A.* 2014;111(24):8901-6.
35. Jackson AS, Pollock ML, and Ward A. Generalized equations for predicting body density of women. *Med Sci Sports Exerc.* 1980;12(3):175-81.
36. Jackson AS, and Pollock ML. Generalized equations for predicting body density of men. *Br J Nutr.* 1978;40(3):497-504.

37. Akinci B, von Schnurbein J, Araujo-Vilar D, Wabitsch M, and Oral EA. Lipodystrophy Prevalence, "Lipodystrophy-Like Phenotypes," and Diagnostic Challenges. *Diabetes.* 2024;73(7):1039-42.
